# En-Bloc Excision and Autogenous Fibular Reconstruction for Giant Cell Tumor of Distal Radius in a Developing Country: Lessons Learned

**DOI:** 10.7759/cureus.81716

**Published:** 2025-04-04

**Authors:** Marlon M Mencia, Laeshelle S Basanoo, Raakesh Goalan

**Affiliations:** 1 Department of Clinical Surgical Sciences, The University of the West Indies, St Augustine Campus, Saint Augustine, TTO; 2 Intensive Care Unit/Anaesthetic Department, Sangre Grande Hospital, Sangre Grande, TTO; 3 Department of surgery, Sangre Grande Hospital, Sangre Grande, TTO

**Keywords:** complications, distal radius, en bloc excision, fibular reconstruction, giant cell tumor, low-resource

## Abstract

Giant cell tumors (GCT) are rare, locally aggressive tumors with a high rate of recurrence after treatment. Most tumors occur around the knee joint, but the distal radius is the most common location in the upper limb. While several treatment options have been proposed; management remains controversial. We present the first two cases of GCT of the distal radius treated with en-bloc resection and autogenous fibula grafting at our institution. The challenges that we encountered and the lessons learned from these initial cases are discussed, with the aim of guiding other surgeons who may need to manage similar cases in low-resource settings.

## Introduction

A giant cell tumor (GCT) is a benign primary neoplasm of bone, accounting for approximately 5% of all primary bone tumors [1]. Although classified as benign, GCTs are locally aggressive with a tendency to local recurrence and rarely, metastasis [2]. These tumors occur in the third and fourth decades of life and are marginally more common in women than men [3]. The rates of GCTs are reported to be higher in Asian countries than in the Western world [4,5]. In a retrospective analysis of bone tumors, in Trinidad, Ramdass et al. reported that GCTs represented 7.7% of all primary bone tumors, a rate that closely mirrors western statistics [6].

Most GCTs occur at the distal femur and proximal tibia, while the distal radius is the third commonest location representing 10% of all GCTs [7,8]. Despite the lack of strong evidence, some experts believe that GCTs at the distal radius display more aggressive behavior than GCTs at other sites with higher rates of recurrence and malignant change [7,9,10]. Several treatment options have been proposed, including curettage and bone grafting/cement packing; resection and replacement with fibula autograft/allograft; vascularized fibular autograft; and prosthetic replacement [11-16]. Despite the myriad of options, and perhaps due to the unpredictable nature of GCTs of the distal radius, controversy surrounds the ideal treatment. Nevertheless, resection and non-vascularized autologous fibula grafting have emerged as the most popular option, which is particularly suitable for developing countries where this condition is most common [17,18].

In this report, we discuss the complications and lessons learned from treating two cases of GCT of the distal radius with en-bloc excision and autogenous fibula grafting at a rural hospital. This article was previously presented as a podium presentation at the 2023 Caribbean Association of Orthopaedic Surgeons Meeting on October 15, 2023.

## Case presentation

Case 1

**Figure 1:**
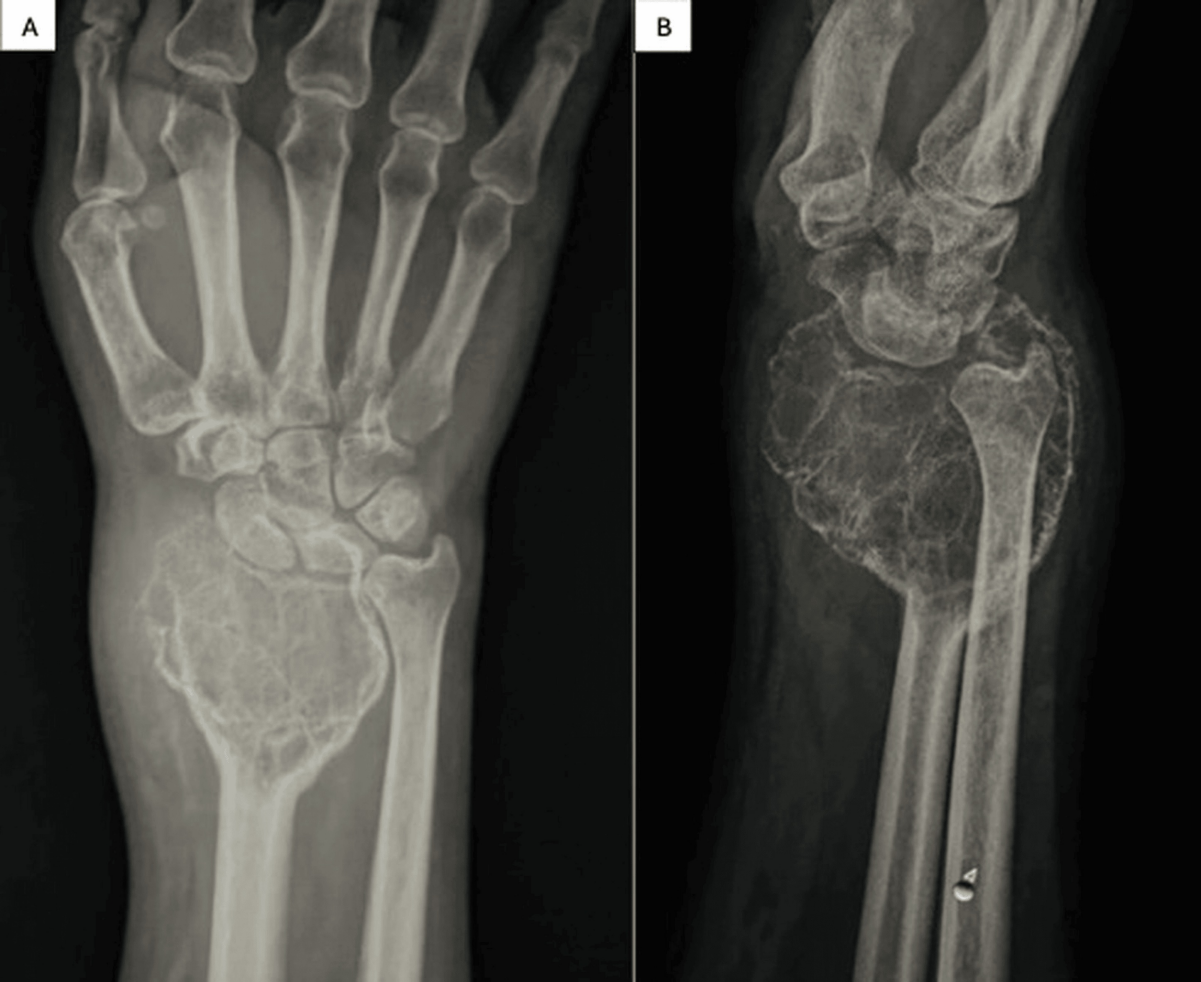
Anteroposterior (A) and lateral (B) views of the wrist showing an expansile lesion of the distal radius with the typical soap-bubble appearance.

A 41-year-old right-handed male farmer presented with a one-year history of progressive pain, swelling, and restricted movement of his right wrist. He reported no previous trauma, and his medical history was unremarkable. Physical examination revealed a hard mass at the distal radius which was mildly tender. There was a generalized limitation of all wrist movements with grade IV power of grip. Radiographs showed an osteolytic lesion that extended into the epiphyseal region with well-defined margins (Figure 1).

**Figure 2:**
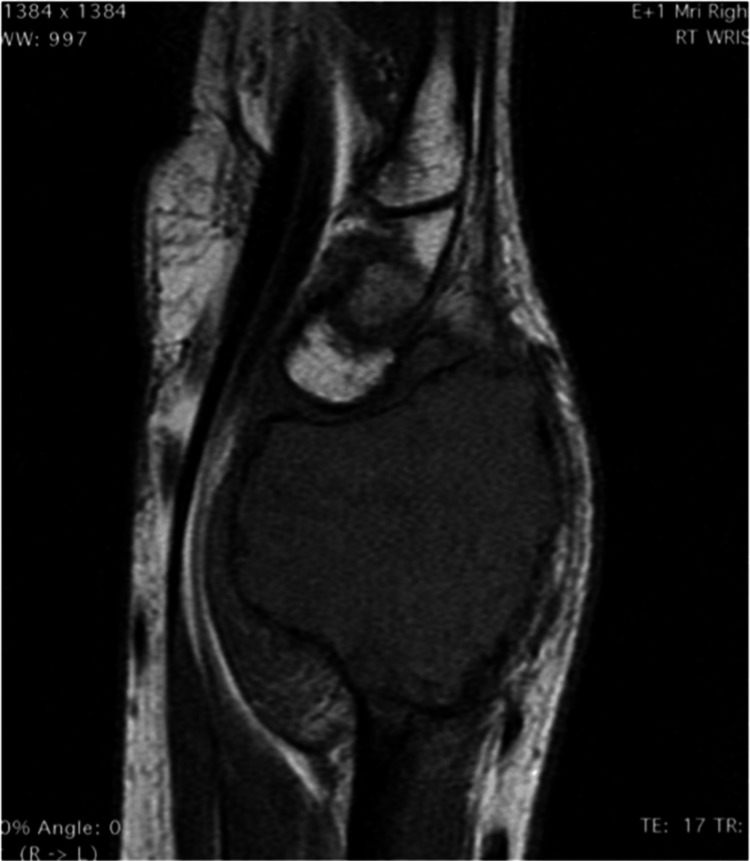
Gadolinium enhanced T1-weighted sagittal MRI image showing a low signal expansile lesion of the distal radius.

An MRI scan revealed evidence of cortical involvement with extension into the soft tissues (Figure 2).

**Figure 3:**
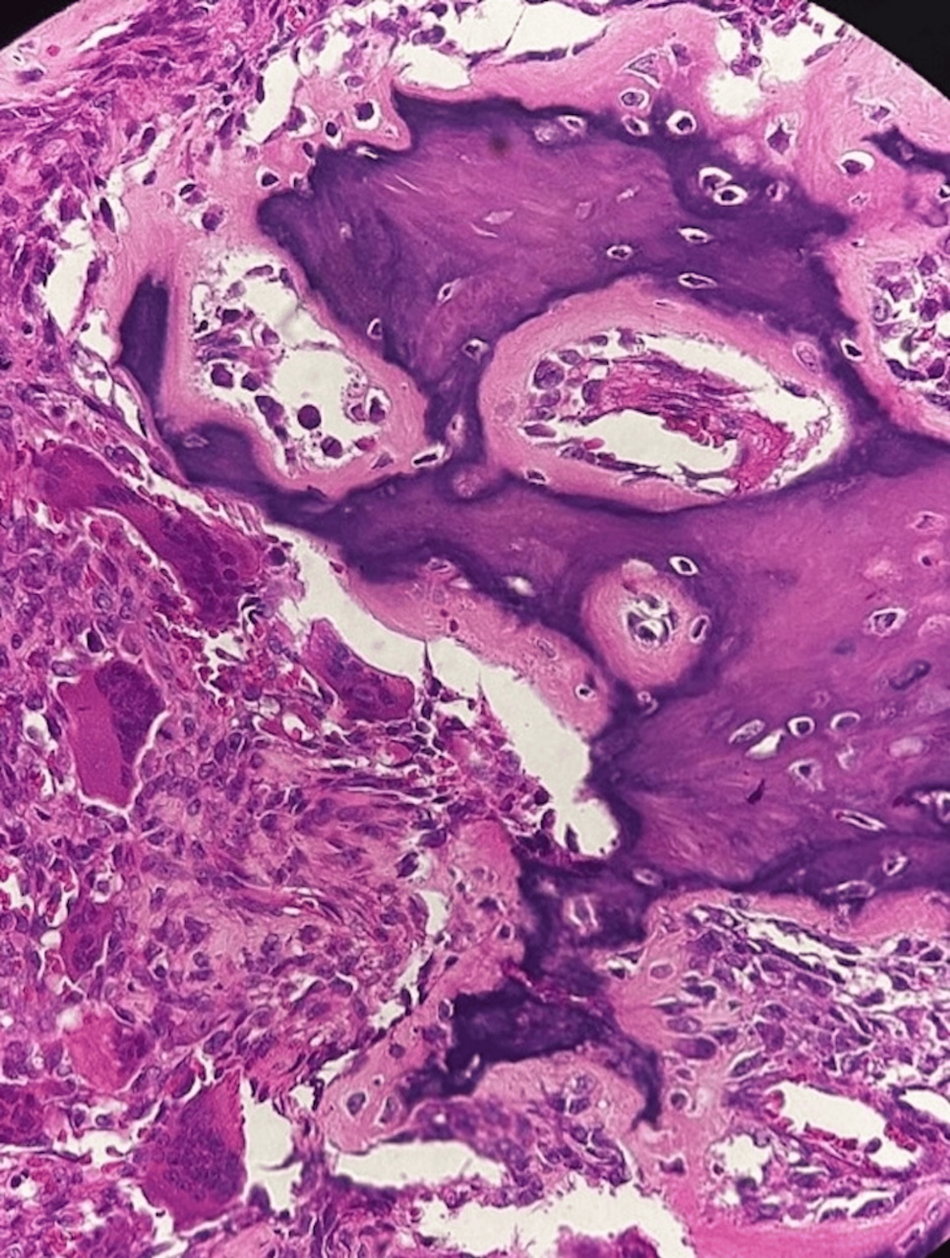
Hematoxylin and eosin (×40)-stained histology slide depicting several multinucleated giant cells embedded within a background of spindle-shaped mononuclear stromal cells.

Biopsy of the lesion exhibited a high density of multinucleated giant cells and spindle cells within a collagenous matrix, which was consistent with a diagnosis of GCT of bone (Figure 3).

A chest CT showed no evidence of distant spread and the patient was assessed as having a Campanacci III GCT of the distal radius.

**Figure 4:**
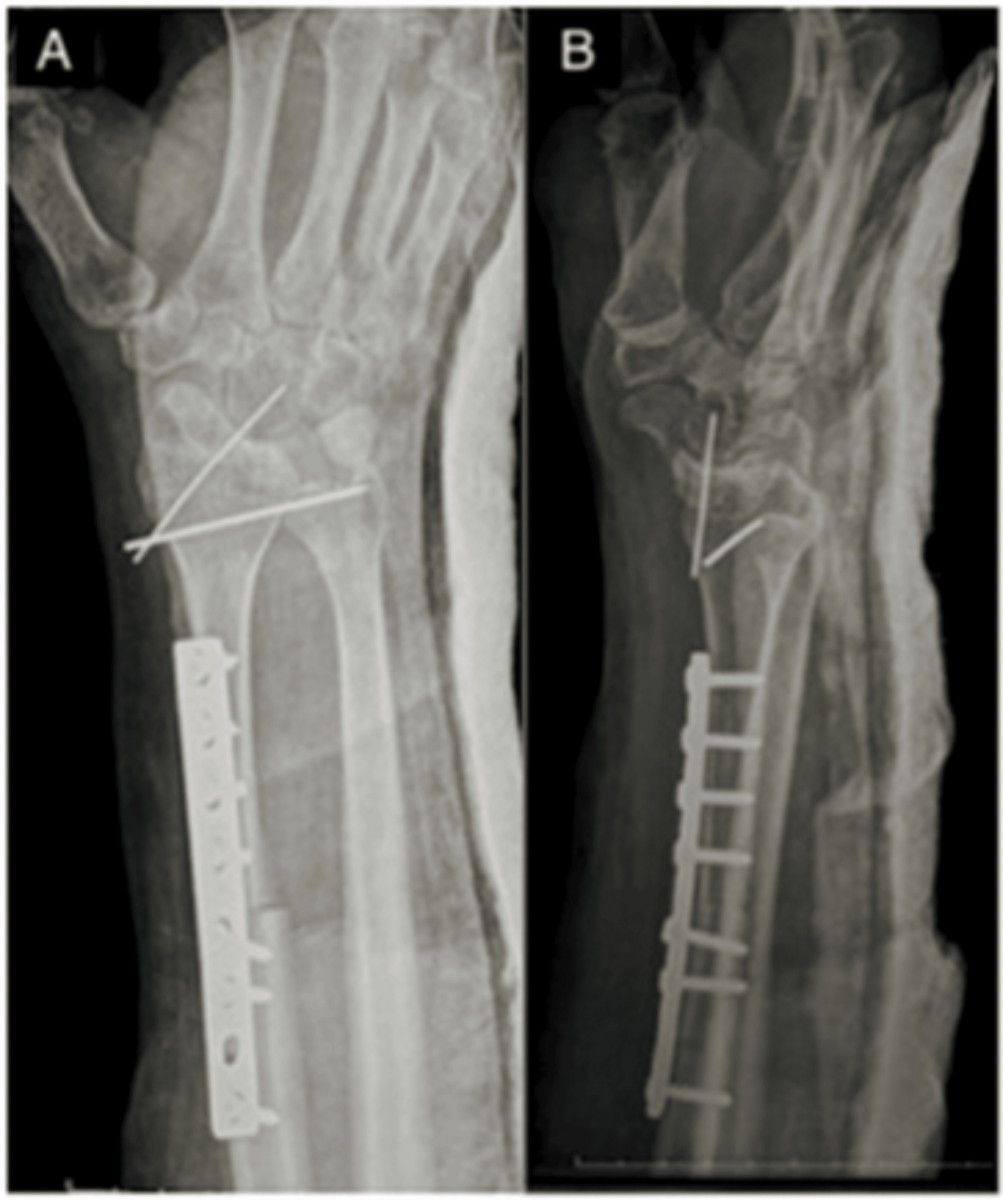
Immediate anteroposterior (A) and lateral (B) postoperative radiographs.

The patient was informed of the diagnosis and gave informed consent to undergo tumor excision and autologous fibula grafting. The patient tolerated the surgery well and postoperative radiographs were satisfactory (Figure 4).

**Figure 5:**
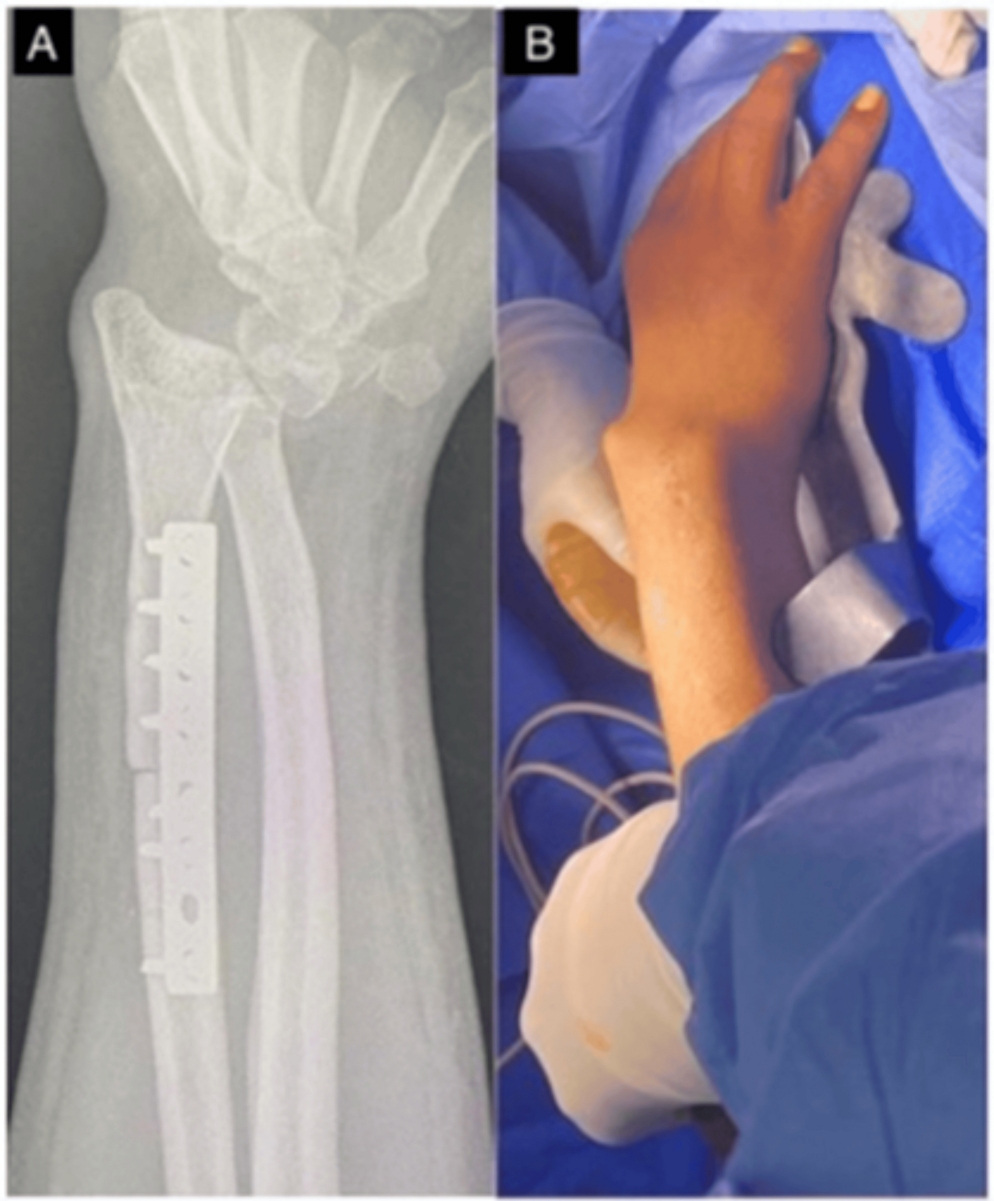
Lateral radiograph (A) and intraoperative clinical photograph (B) showing wrist subluxation.

However, two months later he developed worsening wrist pain, and radiographs revealed significant wrist subluxation (Figure 5).

**Figure 6:**
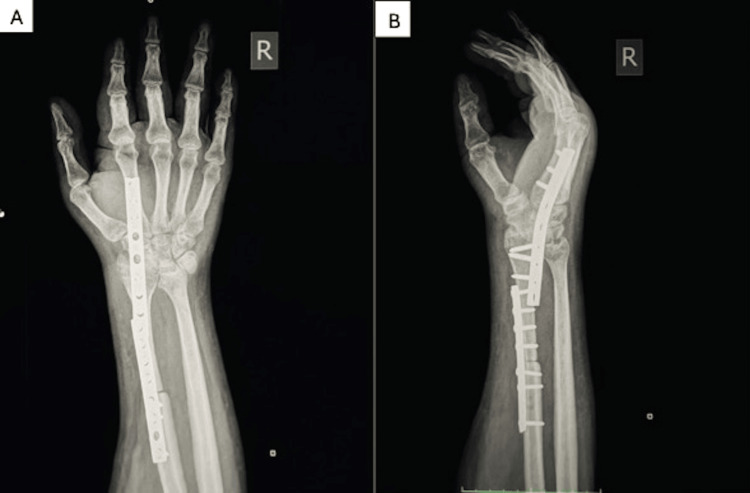
Anteroposterior (A) and lateral (B) views showing the wrist arthrodesis.

After discussing treatment options, we concluded that arthrodesis would be appropriate. Using a standard direct dorsal approach, we contoured and applied a 9-hole 3.5 mm dynamic compression plate (DCP) across the wrist joint to achieve stable fixation in a functional position (Figure 6).

The patient recovered well and has since returned to light farming duties.

Case 2

**Figure 7:**
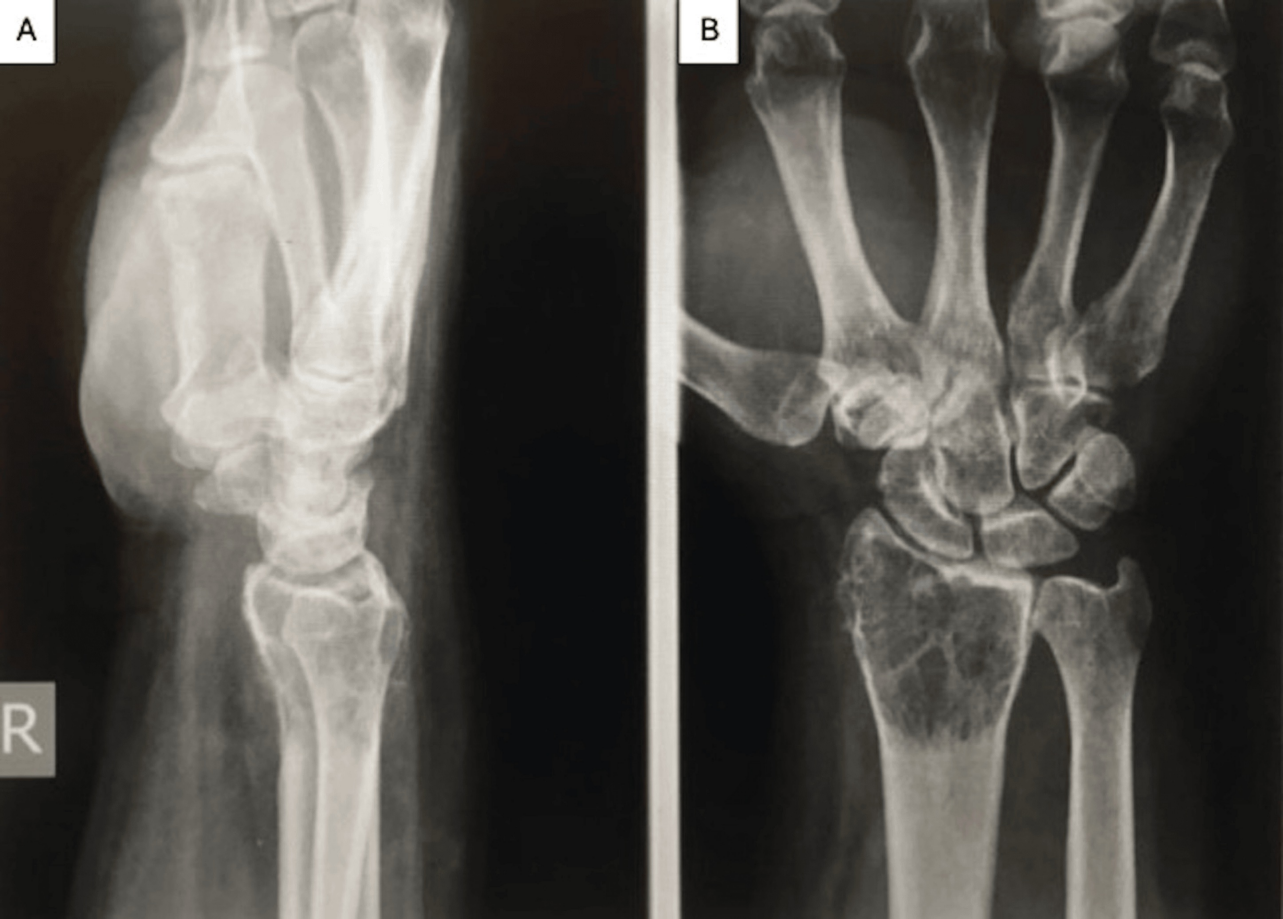
Anteroposterior (A) and lateral (B) views of the wrist showing a well-circumscribed soap-bubble lesion of the distal radius.

A 49-year-old right-handed farmer presented with a slow-growing mass on his dominant wrist over a two-year period. While the mass was painless and did not affect his function, its increasing size was alarming to the patient and prompted him to seek medical advice. Plain radiographs and an MRI were suspicious for a GCT of bone (Campanacci II) which was confirmed on biopsy (Figure 7).

**Figure 8:**
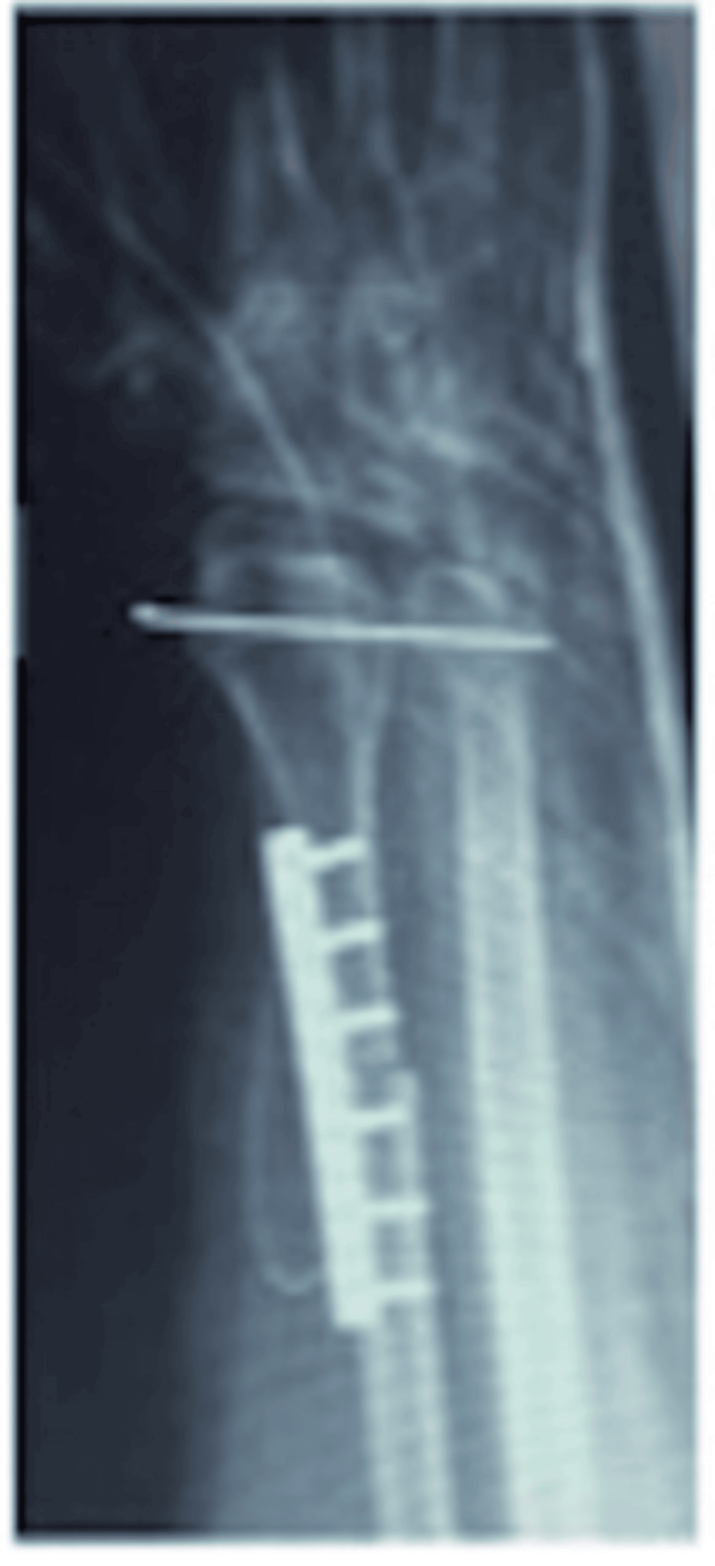
Immediate postoperative radiograph.

Staging investigations showed no evidence of spread, and the patient underwent excision of the distal radius with autogenous fibula grafting (Figure 8).

**Figure 9:**
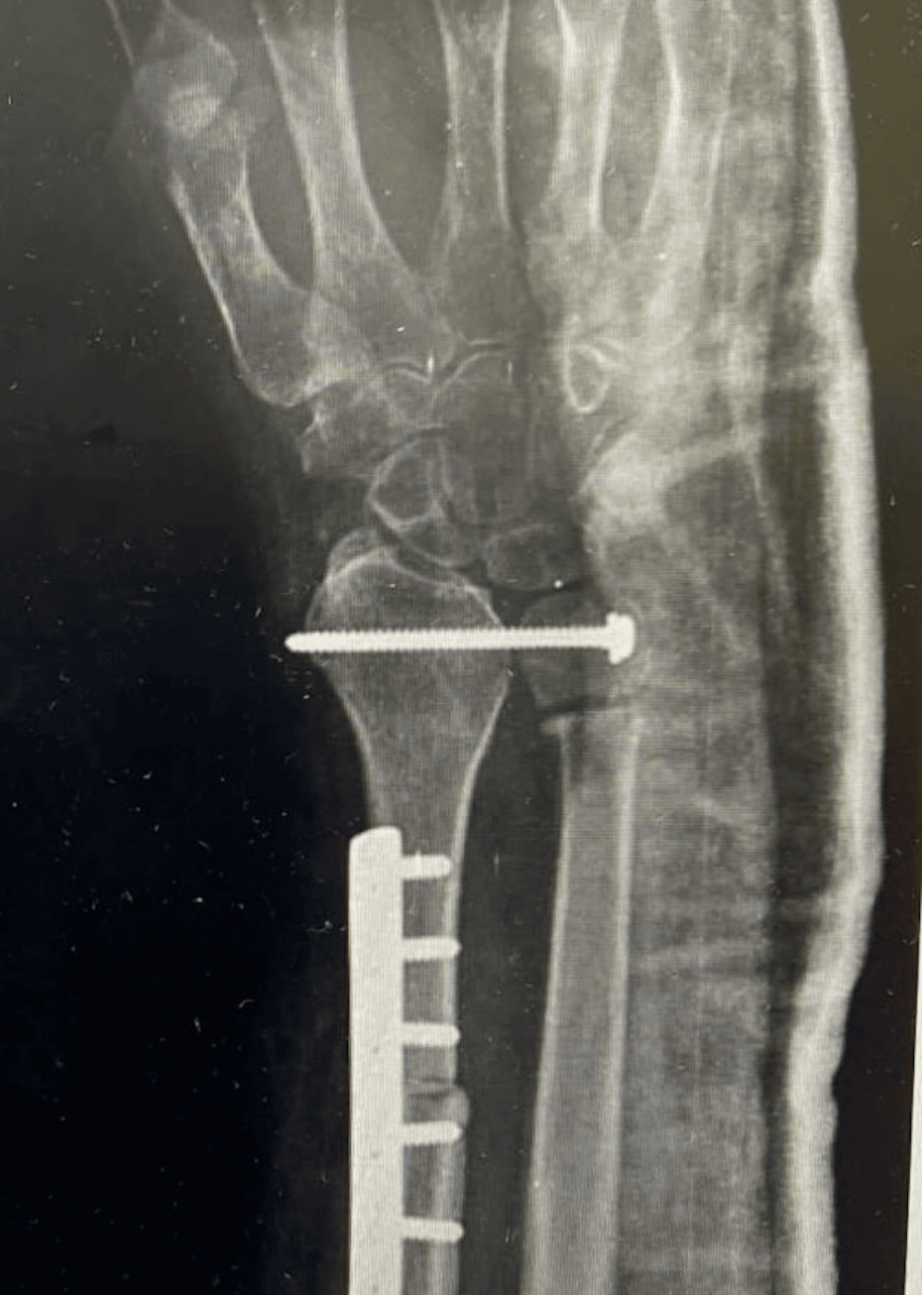
Anteroposterior radiograph of the wrist post-Suavé-Kapandji procedure.

Three months after surgery, radiographs revealed a widening of the distal fibulo-ulnar joint and severe wrist pain. We considered the treatment options and decided on a Suavé-Kapandji procedure to address the instability (Figure 9).

The postoperative period was uneventful, and the patient reported better wrist function and less pain.

Table 1 shows the functional assessment of both patients at a minimum one-year post-surgery follow-up.

**Table 1:** Quick-DASH and EQ-5D scores at a minimum one-year post-surgery follow-up. Quick-DASH (Disabilities of the Arm, Shoulder, and Hand), EQ-5D EuroQol- 5 Dimension

	Quick-DASH	EQ-5D
Case 1	43.2	0.660
Case 2	15.9	0.819

Surgical technique

**Figure 10:**
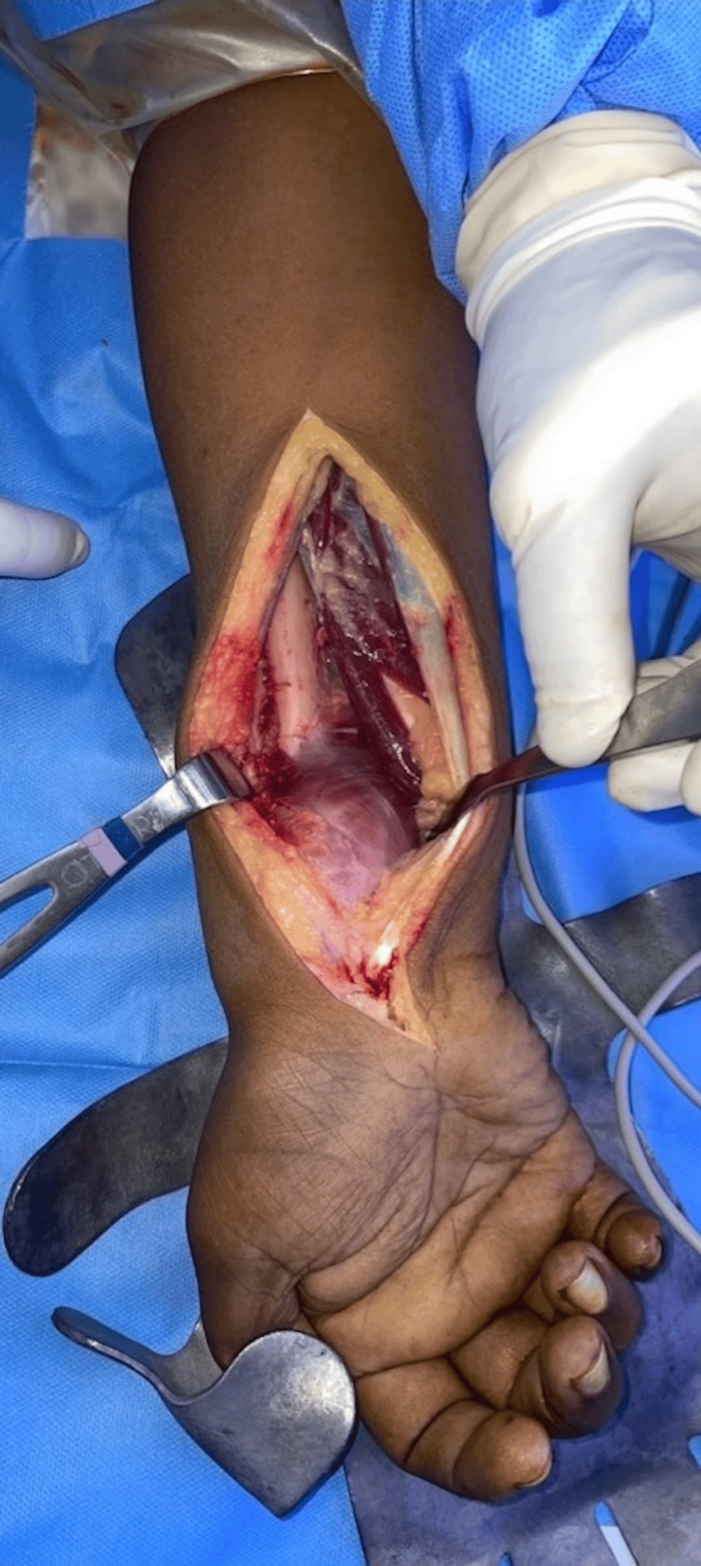
Intraoperative clinical photograph of the giant cell tumor exposed through a standard Henry’s surgical approach.

Under general anesthesia, the affected forearm and ipsilateral leg were prepped and draped appropriately. A pneumatic tourniquet was applied to the arm but not inflated, and in both cases, it was not required during the operation. The forearm was approached using Henry’s surgical approach, incorporating the biopsy tract. Exposure of the radius remained extra periosteal to avoid contamination of the soft tissues with tumorous tissue (Figure 10).

Osteotomy of the radius was planned approximately 3-5 cm proximal to the level of bone involvement on preoperative investigations, with the total length of the resected radius being not less than 10 cm. The technique of osteotomy used a sharp micro-oscillating saw with normal saline to reduce the risk of thermal necrosis. We attempted to preserve the radiocarpal ligaments if they were not involved in the disease, and the tumor bed was bathed in dilute hydrogen peroxide for three minutes and then irrigated with 0.9% normal saline.

**Figure 11:**
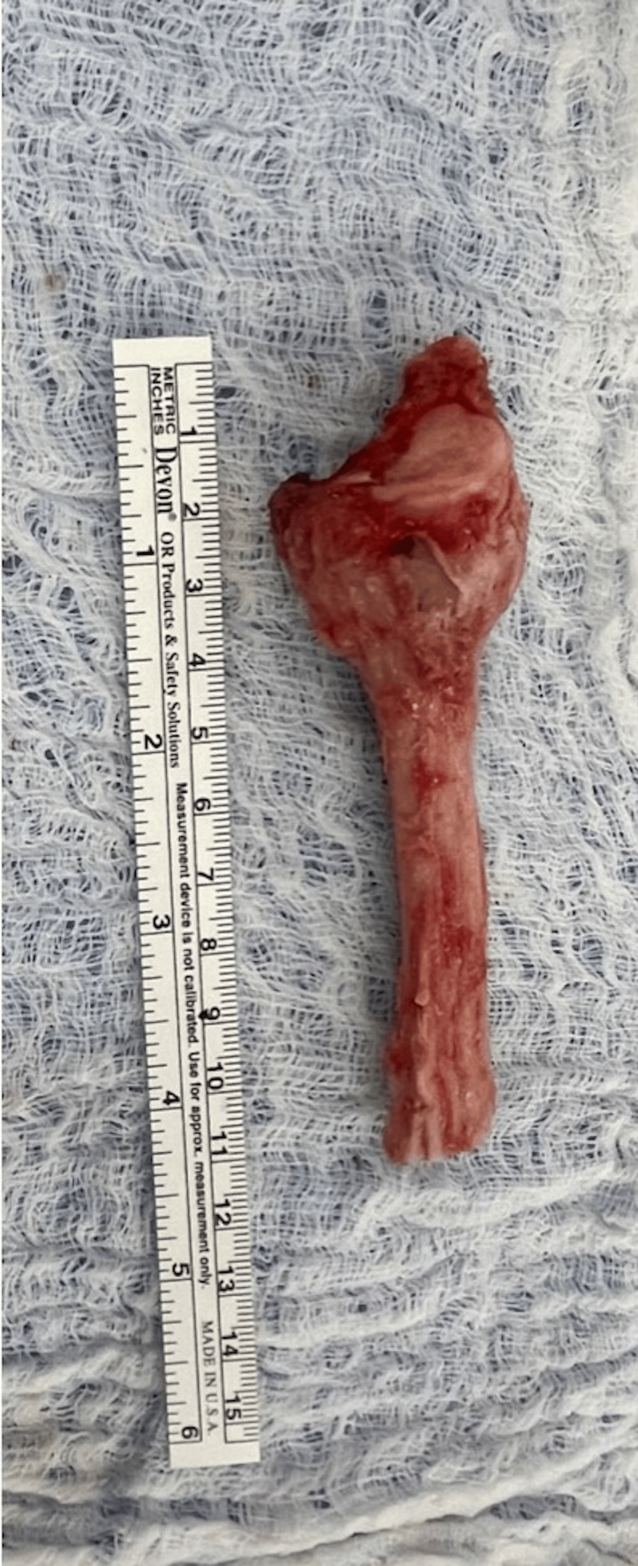
Intraoperative clinical photograph of the harvested proximal fibula.

We next turned our attention to the ipsilateral fibula. Using a direct lateral approach, the peroneal nerve was identified at the level of the fibular neck and protected using a vascular sling. The nerve was dissected proximally and distally to allow some mobility taking care to avoid devascularization. The fibula thus exposed was measured approximately 5 mm longer than the resected radius to allow for compression at the radio-fibula graft site (Figure 11).

**Figure 12:**
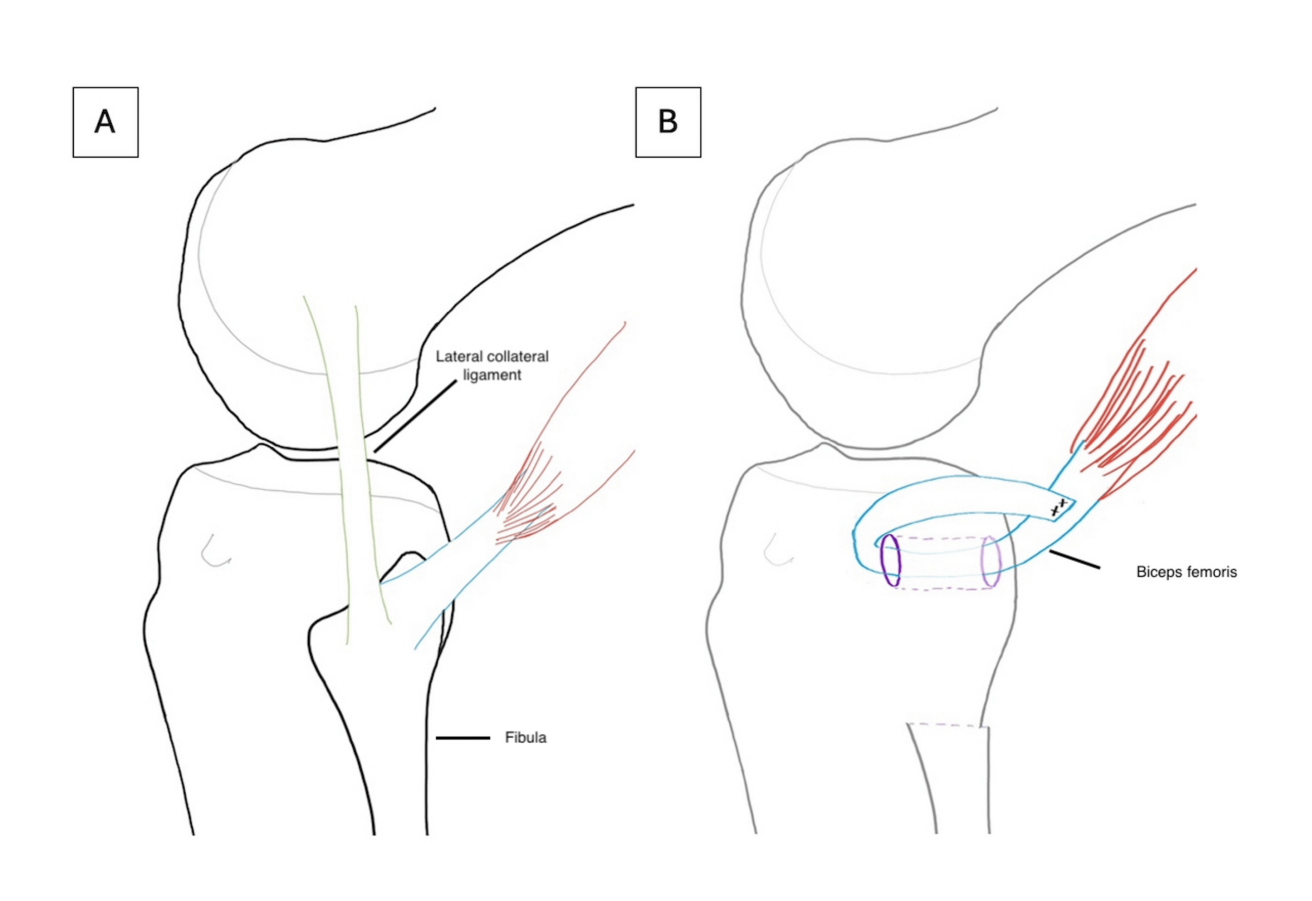
Illustration of the biceps femoris tendon rerouted through a drill hole in the lateral tibial condyle and sutured onto itself after harvesting of the proximal fibular and lateral collateral ligament.

The fibula was osteotomized similarly as described previously. A 1 cm cuff of the lateral collateral ligament (LCL) was resected with the fibula to be used for reconstruction at the fibulo-carpal joint. The biceps tendon was rerouted through a drill hole in the proximal tibia and sutured back onto itself with 2 Vicryl® (Ethicon Ltd., Edinburgh, UK.) (Figure 12).

The common peroneal nerve was placed in the bed of the resected fibula and covered with the peroneal muscle bellies. After achieving hemostasis, the wound was closed in layers.

**Figure 13:**
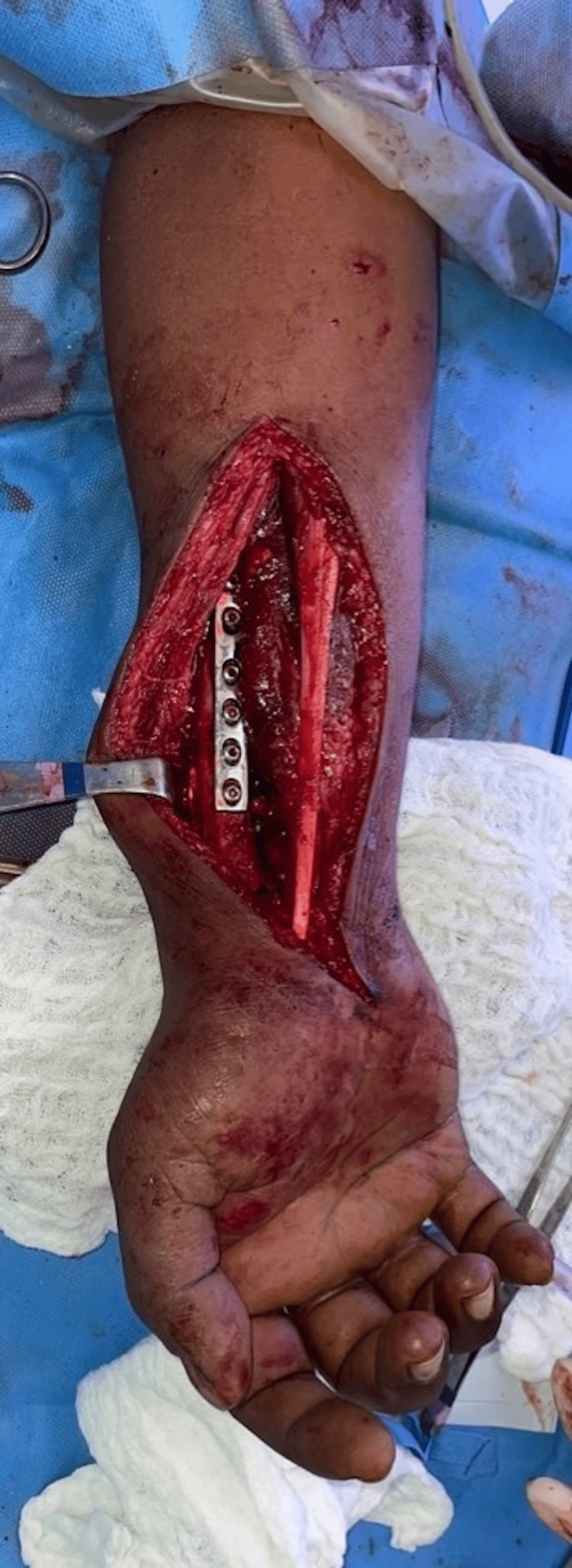
Intraoperative clinical photograph with the harvested fibula, fixed in place using a dynamic compression plate.

The fibula graft was then placed in the bed of the radius, with its distal extent positioned approximately 5 mm beyond the tip of the ulnar styloid and stabilized using smooth k-wires across the fibulo-carpal and fibulo-ulnar joints. This allowed us to adjust the fibular length to restore soft tissue tension while maintaining the position at the distal fibular articulations. Having determined our final length, the fibula was secured to the radius using an 8 or 9-hole 3.5 DCP (Figure 13).

Morselized fragments of the fibula were packed around the compression site to encourage healing. We then attempted to repair the native radiocarpal ligaments to the LCL, with the goal of further stabilizing the fibulo-carpal joint. The wrist was gently moved through a range of movements to estimate gross stability before the wound was closed in layers, and an above-elbow back slab was applied in mid-pronation.

**Figure 14:**
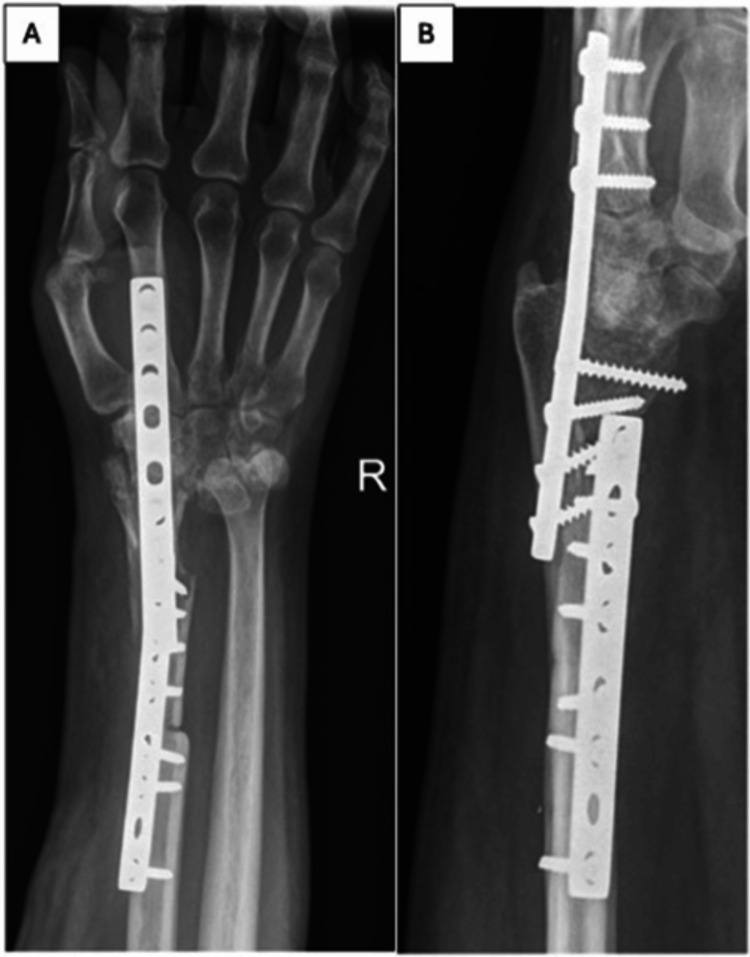
Anteroposterior (A) and lateral (B) views of the wrist (Case 1) showing a solid arthrodesis with no evidence of tumor recurrence.

**Figure 15:**
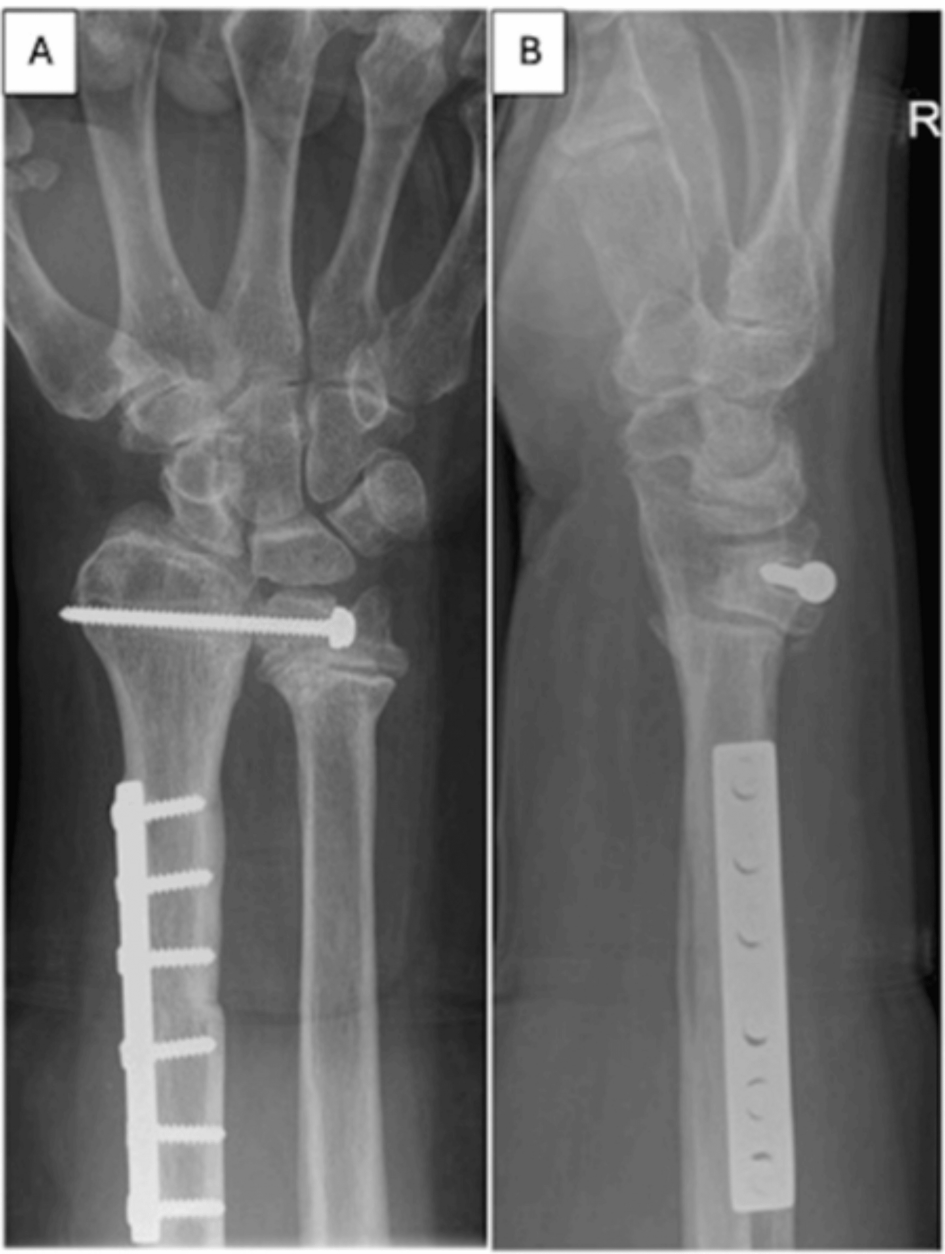
Anteroposterior (A) and lateral (B) views of the wrist (Case 2) with no evidence of tumor recurrence.

The back slab and k-wire were removed at two months, and the patient was referred for gentle wrist exercises to improve movement and strength. Follow-up in the clinic was at six months, 12 months, and annually thereafter. Radiographs were taken at one year to assess for tumor recurrence, union, and other graft-related complications (Figures 14-15). 

## Discussion

GCTs of bone are locally aggressive lesions that frequently show biological behavior inconsistent with radiological or histological evidence [14,19]. When found at the distal radius, GCTs are more aggressive and have a higher likelihood of recurrence and malignant transformation compared to other sites [7,10]. The management of GCT is affected by several factors such as tumor location, size, grade, and individual patient characteristics. The primary focus of treatment is oncological disease control, preservation of function, and minimizing recurrence from the other tenets of care [20]. Surgical management can be broadly classified into either curettage or en-bloc resection and reconstruction [21]. In developing countries, patients often present with advanced-stage lesions that are no longer suitable for curettage [17]. These characteristics make en-bloc excision and autogenous fibula grafting an attractive option for definitive treatment in low-resource countries [7,11,22-24]. Walther, in 1911, was the first to describe the use of a free non-vascularized proximal fibula graft to replace the resected distal radius [25]. Since then several studies have reported good outcomes using this method [5,26-28]. A 2022 systematic review of the reconstructive techniques following wide resection for GCT of the distal radius reported that reconstruction with a non-vascularized fibula provided the best results with low morbidity [29]. However, this approach presents a significant reconstructive challenge for surgeons and is associated with a high complication rate [20,24,30-33].

Successful healing of a non-vascularized bone graft relies on the process of creeping substitution. This biological process progresses through three stages until finally, the graft has completely united with the host bone, functioning as a structural and biological part of the skeleton. Factors affecting this process include the size and quality of the graft, mechanical stability, and vascularity of the recipient site. If revascularization is inadequate the graft may fail to integrate, leading to delayed healing or non-union. Non-union at the host-graft junction is a common complication in several reports of GCT of the distal radius treated by non-vascularized fibula graft with rates from 16% to 50% [17,18,34]. Saraf and Goel in a retrospective study of 42 patients treated, by dynamic compression plating resulted in the lowest non-union rate (33%) compared with intramedullary nail (43%), and a single compression screw through a step cut (50%) [17]. Similar results were reported in a smaller study which showed that dynamic compression plating, reduced the mean time to union compared with an intramedullary rod (5 months vs 8 months) and was further reduced to 4 months if cortico-cancellous graft was used at the graft-host junction [18]. Rapid union is important as it reduces the postoperative immobilization period leading to better functional results. Both of our patients were treated using a DCP and healed uneventfully at 12 weeks. With the evidence available we now recommend the use of cortico-cancellous grafting at the graft-host junction to further reduce this period.

Wrist instability is a common complication following reconstructive surgery and may occur at the fibulo-carpal joint, fibulo-ulnar joint, or both. The incidence of this complication ranges between 10% and 62.5% [17,18,31,34,35]. In a study of 24 cases by Saikia et al., wrist subluxation was the most common complication occurring in 10 (41.7%) patients [5]. A similar rate of wrist instability (47.6%) was reported by Saraf and Goel. Of note, six patients classified as having severe instability required surgical intervention while the other 14 patients with mild instability were treated conservatively. Conversely, Aithal and Bhaskaranand investigated 30 cases of GCT of the distal radius treated with a non-vascularized, autogenous fibula graft and reported only three (10%)cases of wrist instability [31]. Wrist stability depends on both the skeletal congruity of the joint surfaces and the integrity of the adjacent ligaments. However, the inherent incongruence of the fibulo-carpal joint together with scanty ligaments following wide resection, make wrist instability a frequent complication. Fortunately for most patients with this complication, the symptoms are mild and treatment with a wrist brace is usually sufficient [31,34,35]. However, in cases of severe wrist instability with significant symptoms, further surgery is usually necessary. In our series, both patients experienced wrist instability that required surgical intervention. In one case, severe symptomatic fibulo-carpal subluxation required arthrodesis, while in the other case, fibulo-ulnar diastasis was treated with the Sauvé-Kapanji procedure.

Having to reoperate on both patients for wrist instability was an unforeseen challenge, and while this development is indeed disheartening, it presents an invaluable opportunity to gain insightful lessons from our initial experience. We now appreciate that proximal stability at the fibulo-radial junction is critical to stability at the wrist. Several studies have demonstrated that more rigid fixation at the fibulo-radial junction with plates has resulted in less wrist instability and better overall function [17,18,31]. Furthermore, temporary k-wire fixation at the wrist should not be removed early and the final wrist position should allow good hand function in cases of unintended wrist arthrodesis [31]. In light of these facts, we now recommend that k-wires are removed at three months and a functional below elbow, removable brace is used for a further three months.

## Conclusions

Non-vascularized fibular autograft reconstruction for GCT of the distal radius is associated with a relatively high complication rate, with symptomatic wrist instability being the most common and significant issue leading to revision surgery. However, refinement of surgical techniques, especially those aimed at enhancing wrist stability can help mitigate this risk and improve overall outcomes. Importantly, despite these complications, most patients report high levels of satisfaction with their functional results. In cases where the initial reconstruction fails, salvage procedures such as wrist arthrodesis have demonstrated good clinical outcomes, offering patients a reliable safety net.

In summary, non-vascularized fibular autograft remains a valuable reconstructive option for distal radius GCT, especially in developing countries. With improved surgical techniques focusing on wrist stability, this method offers a balanced combination of accessibility, reliability, and satisfactory functional outcomes.
